# Computational vaccine development against protozoa

**DOI:** 10.1016/j.csbj.2025.06.011

**Published:** 2025-06-04

**Authors:** Omar Hashim, Isabelle Dimier-Poisson

**Affiliations:** a Lovaltech, Tours, France; b BioMAP, UMR ISP 1282 INRAe, Université de Tours, France; c Department of Pharmacology, University of Gezira, Wad Medany, Sudan

**Keywords:** Computational vaccinology_1_, Protozoa_2_, Immuno-informatics_3_, Vaccination_4_, Multi-epitope peptide vaccines_5_

## Abstract

Protozoan parasites remain a major global health and economic burden, particularly in low- and middle-income countries. Conventional strategies such as chemotherapy and vector control face growing limitations due to resistance, toxicity, and implementation challenges. Vaccination represents a sustainable solution, but the complexity of protozoan life cycles and antigenic diversity has hindered vaccine development. Computational vaccinology offers innovative tools to overcome these barriers, combining immuno-informatics, reverse vaccinology, and artificial intelligence to accelerate the identification of immunogenic epitopes and streamline vaccine design.

This review explores the current landscape of computational vaccine development against protozoa, highlighting advances in epitope prediction, population-specific vaccine design, and digital twin technologies. Applications include multivalent vaccines targeting conserved antigens across species, personalized formulations based on host immunogenetics, and the emerging use of protozoan vectors in cancer immunotherapy. Despite these promising avenues, significant challenges remain, particularly the need for robust experimental validation, improved delivery systems for short peptides, and greater acceptance of in silico methods by the broader scientific community.

We argue that integrating computational tools with experimental immunology, high-throughput genomics, and translational research will be the key to developing safe, effective, and broadly accessible vaccines against protozoan infections. This convergence of disciplines has the potential to not only address neglected tropical diseases but also to establish new paradigms in precision vaccinology and immunotherapy.

## Graphical Abstract

**Figure:**
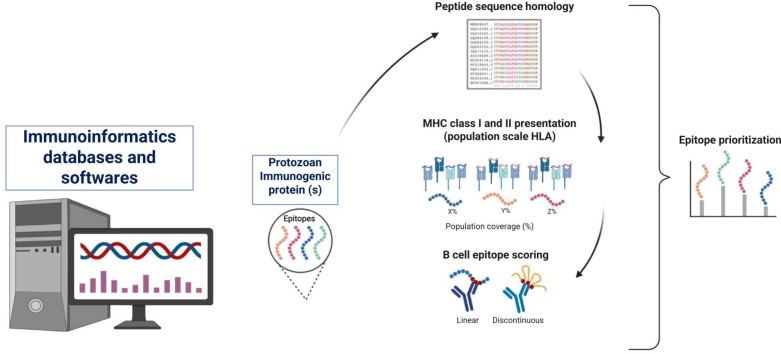

## 1 Introduction

Protozoan parasites present a significant public health burden, especially in low and middle-income countries. Diseases such as malaria, leishmaniasis, amoebiasis, and Chagas disease contribute substantially to global morbidity and mortality, often compounded by underreporting and weak surveillance systems [1], [2]. Conventional control methods including chemotherapy and vector control remain essential but are increasingly limited by drug resistance, toxicity, logistical constraints, and high costs [3].

Vaccination offers a long-term, sustainable strategy to mitigate the burden of protozoan diseases. However, the development of effective vaccines against protozoa remains challenging due to the complexity of parasite life cycles, high antigenic variability, and immune evasion strategies. In this context, immuno-informatics has emerged as a promising approach to accelerate rational vaccine design. This rapidly evolving field builds on advances in computational vaccinology already applied to bacterial and viral pathogens, for instance, in the rapid design of COVID-19 vaccines through antigen prediction and epitope mapping. Although still underutilized in protozoan vaccinology, computational strategies hold great potential for improving the speed, specificity, and efficacy of vaccine development. Malaria remains the most prominent example of a protozoan disease with global consequences. According to the World Health Organization (WHO), 249 million malaria cases were reported across 85 countries in 2022, an increase of five million from the previous year [4]. The economic burden is considerable, exceeding 4.3 billion USD in 2016 when accounting for prevention, diagnosis, treatment, hospitalization, and indirect costs such as absenteeism. Encouragingly, the 2024 WHO World Malaria Report noted that 17 countries had introduced WHO-recommended malaria vaccines into their national immunization programs. However, the report also highlighted persistent inequalities in global malaria control and called for strengthened international collaboration to meet elimination targets [5]. Beyond human health, protozoan diseases also have a major economic impact in veterinary medicine. Livestock diseases such as toxoplasmosis, neosporosis, babesiosis, and coccidiosis cause considerable losses in productivity and trade [6], [7]. Many protozoan infections are zoonotic, with pathogens such as *Toxoplasma gondii*, *Trypanosoma cruzi*, *Leishmania spp*., and *Babesia spp.* affecting both animals and humans. These diseases are often underestimated in global health strategies due to insufficient data, particularly in low-income settings [8], [9].

## 2 Challenges in preventing and treating protozoan infections

### 2.1 Vector control

Many protozoan diseases are vector-borne, prompting vector control strategies as the first line of prevention measures. These strategies include the use of insecticides against larval and adult stages of vectors, mosquito nets and topical repellents. While demonstrably effective in reducing disease burden, their impact remains limited by the emergence of insecticide-resistant vector populations, resource constraints, and the inconsistent implementation in regions most affected by protozoan diseases [8].

### 2.2 Chemotherapy

The primary treatment for protozoan diseases relies on chemotherapeutic agents. However, drug resistance poses a growing challenge. For instance, *Plasmodium falciparum* has developed resistance to nearly all antimalarial drugs, including chloroquine and artemisinin derivatives [10], [11]. Similarly, leishmaniasis treatment faces similar obstacles related to toxic side effects and emerging drug resistance [12]. The rapid emergence of multidrug-resistant parasites and the lack of suitable drug targets continue to hinder control measures [13]*.* In the veterinary domain, the overuse of chemotherapeutics has led to increased drug residues in animal products, posing risks to public health [7]. These challenges highlight the necessity of vaccines as a long-term solution. For example, studies on alternative therapies, including combination treatments, have shown promise but require further validation [12].

Historically, massive vaccination campaigns have played a crucial role in controlling and reducing the mortality of several bacterial and viral infections. Smallpox was eradicated due to vaccination while diseases like measles, polio, and guinea worm are on the way to being fully controlled [14].

## 3 Traditional vaccines against protozoa infections

The development of human vaccines against protozoan infections lags significantly behind their bacterial and viral counterparts [15]. Among the various protozoan diseases affecting humans, only two vaccines RTS/S01 [16] and R21-Matrix-M [17], [18] against *P.falciparum* have been approved by the WHO in 2021 and 2024, respectively [19]. However, the urgent need for vaccines against other protozoal diseases remains unmet.

Malaria, the most prevalent and economically devastating human protozoan disease, serves as a focal point for vaccine development efforts. *Plasmodium falciparum*, responsible for over 90 % of global malaria cases, is the primary target [20], [21]. To combat this, the WHO aims to eradicate malaria in 35 African countries by 2030. Achieving this goal requires vaccines capable of providing 75 % protection with efficacy lasting over two years. Current efforts focus on multi-stage vaccine designs addressing the pre-erythrocytic, erythrocytic, and sexual stages of the parasite lifecycle [22]. Currently, about 191 clinical trials related to malaria vaccines are registered on Clinical Trials.gov. These studies involve efforts to improve existing vaccines and to develop novel candidates targeting various lifecycle stages of the parasite. Circumsporozoite protein (CSP) remains the most studied antigen in the pre-erythrocytic stage, and forms the basis for the two WHO approved vaccines [22]. For the erythrocytic stage, merozoite surface protein (MSP) and RH5 are the key antigens under investigation [23], [24], while Pfs230, Pfs25, and Pfs48 are the most studied sexual stage antigenic targets for transmission blocking vaccines [25], [26]. However, despite some progress, translating animal models data into human clinical efficacy remains a significant challenge highlighting the complexity of malaria vaccine development [27].

Beyond malaria, there is no licensed vaccine against leishmaniasis [12]. Research in this area is scarse with only 23 clinical trials registered against leishmaniasis. Recent preclinical studies utilizing TcTASV proteins from *Trypanosoma cruzi* combined with recombinant baculovirus platforms have shown promise, eliciting robust immune responses such as antigen-specific CD4 + and CD8 + IFNɣ-secreting lymphocytes and achieving over 90 % survival rates in vaccinated animals [28].

The development of vaccines against human protozoal diseases is very limited compared to those targeting veterinary diseases. The relative ease of conducting trials in target animals and less stringent ethical considerations suggest that veterinary vaccine development might offer valuable insights for human protozoan vaccines [29]. Several traditional vaccines were successfully developed against veterinary protozoan diseases, including live attenuated vaccines against bovine tropical theilerosis [30], bovine babesiosis [31], poultry coccidiosis [32], and sheep toxoplasmosis [33]. Additionally, some vaccines utilize defined antigens or extracts, such as those targeting canine leishmaniasis and babesiosis using *Leishmania infantum* cultures and in vitro-cultured canine red blood cells, respectively [31], [34]. A novel nasal vaccine against monkey toxoplasmosis, employing whole parasite proteins, demonstrates the potential for innovative approaches in veterinary applications [35], [36]. In comparison with human diseases, vaccine development for animals has practical advantages such as the ability to perform experiments in the natural host, the possibility to manufacture some vaccines *in vivo*, and lower safety requirements [29].

## 4 Vaccinology in the era of genomics

The genomic era, initiated by the completion of the genome sequence of *H. influenzae* in 1995 has dramatically reshaped biological research [37]. The subsequent release of the human genome sequences [38], [39], along with the release of the genome sequences of many animals and other organisms in the beginning of this century was followed by an explosion of discoveries across multiple fields including vaccinology [37]. Next generation sequencing technologies (NGS) have revolutionized the genomic and genetic research enabling high-throughput analysis and annotation of entire genomes [40].

For protozoan parasite vaccine development, the availability of complete genomic sequences constitutes an inevitable source of information for the development of vaccines, the virtual screening process has allowed the identification and evaluation of the possible vaccine candidates among the pool of antigenic proteins deposited in different databases [41], [42]. Furthermore, the availability of the complete sequences for multiple antigenic protein variants across geographical regions, coupled with sequence alignment databases, has paved the way for immuno-informatics and the revolutionary approach of reverse vaccinology. Additionally, advancements in genomic editing technologies, such as CRISPR-Cas9 have enhanced the functional validation of computational predictions. These tools enable precise manipulation of protozoan genomes, allowing for the targeted disruption or overexpression of genes encoding antigenic proteins to confirm their role in the induction of specific immune responses. Combined with high-resolution structural bioinformatics, these approaches are setting the stage for the next generation of vaccines tailored to protozoan pathogens.

## 5 Computational vaccinology and immuno-informatics

Computational vaccinology integrates a range of bioinformatics tools to support epitope prediction, antigen prioritization, and immunogenicity assessment. Resources such as the Immune Epitope Database (IEDB) offer structured platform for predicting, ranking, and comparing B cell and T cell epitopes based on structural features and predicted immune response. These tools offer valuable insights for the design of highly precise epitope-based vaccines.

Modern computational approaches increasingly leverage artificial intelligence (AI) to enhance prediction accuracy. AI-powered digital twin technologies, for instance, can simulate immune responses in silico to assess vaccine efficacy prior to experimental validation. These tools reduce development time and costs, increase precision in immunological modeling, and support ethical goals such as minimizing animal models experimentation. Computational vaccinology and immuno-informatics, at the intersection of immunology and bioinformatics, rely on specialized databases and tools to support various steps of vaccine development [43], [44]. These include the prediction of B-cell and T-cell epitopes, population coverage analysis, adjuvant selection, and formulation design. The selection of B-cell epitopes is based on several criteria such as surface accessibility (as determined by 3D structure), linear or conformational nature of the epitope, and the ability of activating B cells to produce pathogen-specific antibodies. For T-cell epitopes, the main criterion is their binding affinity to selected MHC molecules. Predictive algorithms such as NetMHCpan, NN-align, Ann-align, and SMM-align are commonly used for this purpose, with established thresholds for MHC I and MHC II binding [45], [46].

Some databases also incorporate filters for pathogenicity and allergenicity helping to eliminate potentially harmful epitopes. Moreover, many platforms now integrate AI-based modules that predict not just binding but also the overall antigenicity of candidate epitopes, enabling more refined selection. Zvyagin *et al.* [47] provide a comprehensive overview of the most widely used immuno-informatics databases and tools, including those for epitope mapping, TCR specificity, and structural modeling. Conservation analysis is another critical step especially for broad-spectrum vaccines. Universal epitopes must be conserved across all known pathogen strains. Sequence repositories such as NCBI and UniProt allow researchers to retrieve the sequences of available variants, which can be aligned using tools like Clustal Omega to identify conserved regions [48].

Reverse vaccinology further strengthens these efforts by enabling re-analysis of antigenic proteins in response to emerging variants. Combined with AI-enhanced immuno-informatics, this iterative approach offers unprecedented opportunities to design next-generation vaccines specifically tailored to protozoan diseases.

In the veterinary field, vaccine development faces unique obstacles due to the limited availability of predictive algorithms for animal MHC alleles. This gap has traditionally led to the use of whole-organism or attenuated vaccines. However, recent advances in computational methods, particularly in machine learning and high-throughput screening are now enabling the development of livestock-specific MHC prediction tools. These approaches open new avenues for the rational design of epitope-based vaccines adapted to animal immune responses, an area with strong relevance to zoonotic disease control and translational vaccine research

## 6 Population vaccinology

Traditional live-attenuated vaccines played a pivotal role historically in the fight against pathogens and even eradication of some diseases like smallpox and rinderpest [49]. However, these vaccines are not without limitations including vaccination failure, possible pathogenicity especially in immunocompromised patients and a lack of population-specific considerations. The “one vaccine fits all” approach received increasing criticism due to variability in vaccine responses and adverse events across different populations, influenced by genetic diversity and environmental factors.

Population vaccinology addresses these limitations by tailoring vaccines to specific populations, leveraging tools that analyze prevalent MHC alleles, particularly MHC I and MHC II. These alleles significantly influence vaccine protection efficacy by dictating the presentation and binding of epitopes to immune system molecules [45]. Several databases, such as IEDB and NetMHCpan, leverage immuno-informatics tools to assess epitope binding specificities within different populations. This information can then be used to calculate the percentage coverage of an epitope within a specific population, especially relevant for endemic diseases [45], [50].

**Fig. 1:**
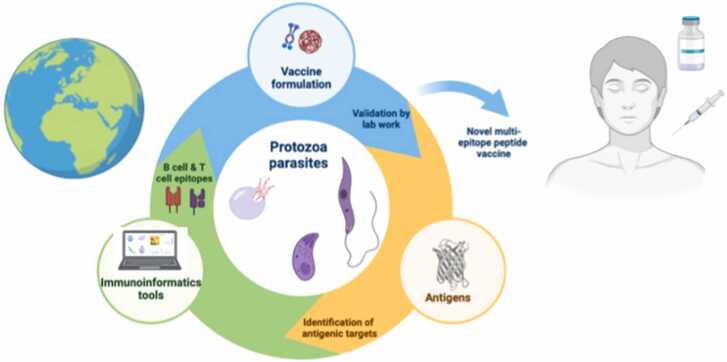
Computational pipeline for the *in silico* design of epitope-based vaccines against protozoan pathogens. The process begins with genome and proteome mining to identify candidate antigenic proteins. These sequences are then screened using immuno-informatics tools for B-cell and T-cell epitope prediction, taking into account MHC binding affinity, antigenicity, allergenicity, and population coverage. Conserved and immunodominant epitopes are prioritized using multiple sequence alignment and conservation analysis across strains or species. Selected epitopes are assembled into multi-epitope constructs, optimized for solubility, stability, and immunogenicity. The final constructs are subject to experimental validation to assess immunogenicity and protective efficacy *in vitro* and *in vivo*.

While population-specific approaches are crucial, the ideal scenario involves identifying conserved epitopes present across all parasite strains, leading to a truly universal vaccine. Existing databases of common MHC alleles serve as a starting point for pinpointing population-specific variants [51]. For example, a malaria vaccine targeting populations in the malaria belt (sub-Saharan Africa, parts of South Asia, and South America) would benefit from a multi-epitope design informed by population immuno-informatics analysis. Primary analysis of several antigens provides a pool of promising epitopes based on their binding to B cell and T cell, but also based on their ability to induce immune responses, while population coverage studies fine-tuning the selection of suitable epitopes for each population in question. Fig. 1 and the graphical abstract describe the central role of computational vaccinology in the process of developing vaccines against protozoa, that includes not only the primary design and conceptualization of antigenic targets but also the validation of all preclinical and clinical stages.

Compared to traditional whole-protein formulations and fusion proteins, the formulation of these epitopes into vaccine is more challenging. B cell epitopes are generally ranging from 12 – 20 aa while most databases set the length of MHC II epitopes at 12 – 18 aa, reflecting the size of their binding domains. These short, often charged epitopes require several chemical modifications to solve the problems of solubility, absorption, and membrane transport. However, these modifications are still sub-optimal and costly which adds to the production cost of the vaccine thereafter. Several strategies are being explored to effectively present short epitopes to immune cells, including the use of virus like particles (VLP), and the use of linkers to join the selected epitopes in sequential manner. The selection of suitable adjuvant is another challenge in this type of vaccines. Multi-epitope peptide vaccines tend to be less immunogenic compared to classical vaccines which necessitates the use of adjuvants to potentiate the effect and to secure long lasting immune responses. The adjuvants also should be compatible chemically with the epitopes as well as with the formulation. Continuous analysis of antigenic proteins used for several vaccines might reshape epitope maps of the antigen and provide insights that help in further improvements. For instance, recent analysis of the CSP protein in the two WHO-approved malaria vaccines revealed the existence of monoclonal antibodies targeting two novel epitopes not currently included in these vaccines, offering avenues for improved vaccine design [52]. This iterative refinement process underscores the transformative potential of computational tools in advancing population-specific and universal vaccine development strategies.

## 7 Preparedness for timely vaccine development depends largely on immuno-informatics and AI

The pandemic of COVID in 2019 highlighted the critical need for fast responses in design and development of vaccines. Since the next pandemic is knocking our doors, the concept of preparedness to the coming pandemics gained a lot of attention in scientific vaccinology communities. The “CEPI 100 days mission” to develop vaccine from conceptualization to market is the next challenge for vaccinologists [53]. Immuno-informatics is the key and the first step for such rapid response, prediction of antigenic proteins of the pathogen in question, identification of the most antigenic epitopes within targeted antigens, selection of the suitable adjuvant, and the best construction and formulation, all these steps could be done in relatively short time and with high degree of precision.

Implementing AI at several key steps of vaccine design, notably for antigen identification by reverse vaccinology, antigen screening in digital twins of immune cells, and antigen production in smart bioreactors has the potential to de-risk this novel form of immunization. Thanks to AI, it’s now possible to create digital twins of cells, a virtual representation of cellulars’ behavior that allow to conduct experiments without physical manipulation. These systems are invaluable assets capable of uncovering new vaccine targets, predicting immune responses, and shedding light on previously unknown aspects of the pathogens and immune system interactions. Implementing AI in industrial production systems has greatly and consistently enhanced efficiency in the process of vaccine development. Smart bioreactors, equipped with sensors and control systems, can continuously monitor and adjust the conditions within the bioreactor to optimize cell growth and antigen production. AI algorithms can predict and preemptively address potential issues, thereby reducing the trial and error typically involved in scaling up bioprocesses. This capability not only shortens the time to market for new vaccines but also ensures that production can swiftly adapt to the demands of emerging health crises.

In conclusion, the synergy between immuno-informatics and AI represents a paradigm shift in vaccine preparedness. By leveraging these technologies, the scientific community can achieve unprecedented speed and precision in vaccine development, ensuring timely responses to future pandemics. This proactive approach not only addresses current global health challenges but also fortifies the world against the inevitable threats of tomorrow.

## 8 Computational vaccinology and protozoa, where are we?

As with classical vaccines, the use of computational vaccinology against protozoa is far behind those for viruses and bacteria. Many studies explored the different antigenic targets in bacteria [54], [55], [56], viruses [57], [58], [59], [60], [61], [62], [63], [64], [65], [66], and fungi [67], [68], [69], [70], [71] while relatively few studies were focused on immunogenic targets of protozoan parasites.

Several studies were conducted to study and evaluate many antigenic proteins throughout the life of *plasmodium falciparum.* Antigenic proteins from the blood stage like RH5 and MSP were analyzed by immunoinformatics tools and predicted an interesting multi-epitope peptide vaccines targeting B cells, CD4^+^ , and CD8^+^ T cells. Some of these studies showed a universal population coverage exceeding 90 % [72]. CSP, one of the most antigenic and extensively studied protein throughout the parasite lifecycle, serves as the foundation for WHO-approved malaria vaccines and a prime target for immunoinformatic analysis. While the NANP repeat region containing B-cell epitopes is highly conserved, CSP is known to be very polymorphic in the C terminal domain containing the T cell epitopes. The epitope mapping of CSP showed very high diversity in sequences from different geographical areas but also within the population of the same area, a study conducted in India showed more than 10 variants of CSP due to mutations in both region 2 region 1 containing the CD4^+^ and CD8^+^ epitopes [73].

Since resident CD8+ memory T lymphocytes play a critical role in long-term protection against malaria [74], [75], and the WHO's target of 75 % protection lasting more than two years underscores the importance of including not only the most immunogenic but also the most conserved epitopes in the C-terminal domain of CSP, this illustrates the pivotal role of computational vaccinology and immuno-informatics

Analysis and evaluation of antigenic proteins already approved for vaccinations continues to be a source of inspiration and new findings. Even though, CSP is well studied and it’s epitope map was published many years ago [76], a recent study on CSP showed two monoclonal antibodies against epitopes in region 2 that are not included in the WHO-approved vaccines [52].

Recent advances in computational vaccinology have led to the prediction of epitope-based peptide vaccines for a range of protozoan pathogens. In *Leishmania donovani*, a multiepitope subunit vaccine targeting conserved epitopic regions of the PrimPol protein was designed using immunoinformatics tools [77]. Similarly, in *Leishmania major*, B-cell-derived T-cell epitopes from the glycoprotein gp63 were identified and formulated with adjuvants targeting TLR-4 to stimulate innate immune responses [78]. A multimeric vaccine design using calcium-dependent protein kinases (CDPK1, CDPK2, CDPK3, and CDPK5) of *Toxoplasma gondii* has been proposed to enhance protection against toxoplasmosis [79], while additional studies have explored novel antigenic targets [80]. Antigenic proteins have also been computationally investigated in other protozoas including *Brugia* [81], *Trypanosoma* [82], and *Cyclospora* [83], although experimental validation remains lacking for most candidates. In contrast, the epitope mapping of CSP from *Plasmodium falciparum* has been extensively confirmed by experimental data [84], [85].

Compared to viruses and bacteria, designing epitope-based peptide vaccines against protozoa is inherently more complex. Protozoan genomes are typically larger, more variable, and equipped with immune evasion strategies that challenge epitope conservation and prediction. While *insilico* tools provide powerful frameworks for antigen selection, adjuvant pairing, and formulation design, experimental validation remains essential. Confirming immunogenicity and protective efficacy *invitro* and *invivo* is crucial for translating computational models into effective vaccines against protozoan infections.

## 9 Limitations and challenges of *in silico* design and computational vaccine development

The development of vaccines based on computational predictions poses unique challenges at multiple levels. These limitations highlight the complexities involved in translating theoretical predictions into effective immunization strategies:

● Epitope Length and Chemistry: Most databases predict short epitopes (8 aa for MHC I and around 15 amino acids for MHC II). These short length peptides are charged (positive or negative) and difficult to formulate in single construct. Chemical modifications are required to enhance solubility, stability, and delivery, which can be both time-consuming and costly.
● Delivery System Integration: Ensuring that all selected epitopes are effectively delivered and presented to their corresponding immune cells (B cells or T lymphocytes) remains a significant challenge. Designing delivery systems that achieve optimal antigen presentation while maintaining the structural integrity of epitopes is an area of active research.
● Adjuvant Compatibility: Multi-epitope vaccines tend to be less immunogenic than traditional formulations, necessitating the use of potent adjuvants. However, identifying adjuvants that are chemically compatible with both the epitopes and the delivery system adds another layer of complexity
● Stability and storage: The stability of peptide-based vaccines under varying storage conditions is a critical concern, especially for vaccines targeting protozoan diseases prevalent in resource-limited settings. Maintaining efficacy at ambient (positive) temperatures is essential for their deployment in tropical regions.
● Reproducibility Across Databases: Different immuno-informatics tools and databases often apply varying algorithms and models, leading to inconsistencies in predictions. Achieving reproducible results across platforms remain a persistent challenge for computational vaccinology.
● Scientific credibility and Trust: Gaining the trust of researchers, particularly traditional immunologists and vaccinologists, is critical for the broader adoption of computational approaches. Skepticism regarding the accuracy and applicability of in silico methods remains a barrier.
● Experimental Validation: The lack of experimental validation for computational predictions significantly limits their acceptance and application. While many bacterial and viral candidates have undergone wet-lab validation, protozoan vaccine predictions are rarely tested in vivo or in vitro, limiting their progression toward clinical use.

## 10 Future prospective

The practical application of computational vaccinology in endemic regions will require a collaborative effort to validate computational predictions and ensure compatibility with local population immunogenetic profiles. Collaborations with local research institutions, clinical research centers, and public health agencies will be essential to support field trials and gather immunological data from diverse populations. Such efforts could significantly reduce the burden of protozoan diseases in affected regions.

### 10.1 Multivalent antiprotozoal vaccines

This approach leverages antigenic similarities across protozoan species. For example, CSP is shared by several *Plasmodium* species, while MSP has homologs in *Plasmodium spp.* and *Toxoplasma gondii*. Identifying such conserved antigens can enable the design of broadly protective, cross-species vaccines.

Multivalent vaccines aim to incorporate epitopes conserved across multiple protozoan species or strains, offering a cost-effective and scalable strategy for simultaneous protection against several pathogens. For example, studies have identified cross-reactive antigens between *Plasmodium falciparum* and *Plasmodium vivax*, which could form the basis for a universal malaria vaccine. Similarly, the SAG1 and MIC antigens of *Toxoplasma gondii* show homology with surface antigens of *Neospora caninum*, suggesting the feasibility of dual-species vaccines.

The development of multivalent vaccines benefits from advanced computational tools capable of identifying conserved antigenic regions across species. Platforms like Clustal Omega enable alignment and analysis of protein sequences to pinpoint universally conserved epitopes. Reverse vaccinology further accelerates this process by focusing on antigenic targets expressed during the most infectious stages of the parasite lifecycle.

Despite their promise, multivalent vaccines face challenges in formulation and delivery. Combining multiple epitopes increases the complexity of vaccine design, necessitating the use of robust adjuvants and delivery systems. Virus-like particles (VLPs) and nanoparticle-based systems have emerged as promising vehicles for presenting multivalent antigens in a manner that enhances immunogenicity while maintaining stability.

Additionally, population coverage analysis is critical for multivalent vaccines, ensuring that selected epitopes are effective across diverse genetic backgrounds. This approach ensures broad-spectrum protection while minimizing the risk of immune evasion.

Future work should focus on experimental validation of computationally predicted multivalent vaccines, evaluating their protective efficacy in both preclinical and clinical settings.

### 10.2 Protozoa-based cancer therapeutic vaccines

Emerging research has shown that some protozoan parasites can elicit potent immune responses, offering potential for cancer immunotherapy. While viruses and bacteria have been widely explored in oncolytic therapy [86], [87], [88], [89], protozoa remain underutilized despite promising early findings.

Protozoan parasites can stimulate broad generalized immune responses. Engineered strains expressing tumor-specific antigens have been developed to increase the tumor specificity of the immune response [90]. For instance, *T. cruzi* engineered to express NY-ESO-1 antigen, a highly immunogenic cancer-testis antigen elicited strong humoral and CD8 + T-cell responses in mice challenged with NY-ESO-1 + melanoma cells, achieving complete tumor rejection [91]. Similarly, attenuated *Plasmodium* sporozoites expressing MAGE-A3 antigen delayed tumor growth and induced MAGE-A3-specific CD8 + responses in lung cancer models [92]. Other studies have demonstrated the anti-tumor properties of *Neospora caninum* and *Toxoplasma gondii*. For example, *N. caninum* tachyzoites injected into murine thymoma models inhibited tumor growth via NK and CD8 + T-cell activation and IFN-γ secretion [93]. Likewise, the recombinant GRA6Nt protein from *T. gondii* has shown potent adjuvant effects in colorectal cancer models [94]. Recent work from our team showed that genetically engineered *N. caninum* and *T. gondii* strains can secrete functional anti-PD-L1 monoclonal antibodies, revealing their capacity to modulate immune checkpoints [95].

Computational vaccinology can enhance this field by facilitating the identification of tumor-associated epitopes, optimizing antigen design, and predicting host immune responses. These tools also offer simulation capabilities via digital twin models to assess tumor-immune interactions, enabling rational engineering of protozoan strains for cancer immunotherapy.

### 10.3 Toward personalized vaccinology

Personalized vaccinology represents a promising frontier in vaccine development. By incorporating individual immunogenomics data into vaccine design, researchers could create vaccines specifically tailored to each person's immune profile. Personalized vaccines would narrow the scope of population vaccinology even further, designing individual-targeted vaccines based on each individual’s unique immunogenomic fingerprint. This strategy is particularly relevant for high-risk populations such as the elderly, immunocompromised individuals, or those with rare HLA variants, computational vaccinology is central to this effort.

In the context of protozoan diseases, leveraging population-specific MHC data to design personalized vaccines could address the unique challenges posed by antigenic diversity and immune evasion. Digital twin technologies further advance this concept by modeling host immune responses *insilico*. These models simulate vaccine efficacy, enabling rapid testing of vaccine formulations without the need for extensive animal trials. When combined with targeted delivery systems such as nanoparticles, this approach could lead to more precise, effective, and safe immunization strategies

While the promise of personalized vaccinology is immense, several barriers must be addressed, including access to high-throughput sequencing technologies, data privacy concerns, and the cost of manufacturing individualized vaccines. Achieving equity in access and scalability will be crucial for the global implementation of personalized vaccination strategies.

In conclusion, future directions in computational vaccinology ranging from multi-valent antiprotozoal vaccines to protozoa-based cancer therapies and personalized vaccine design highlight the transformative potential of integrating immuno-informatics into next-generation vaccine development.

## 11 Conclusion

In summary, computational vaccinology offers significant promise in overcoming the challenges associated with developing effective vaccines for neglected protozoan diseases. Key advances such as reverse vaccinology, AI-driven predictive modeling, and digital twin technology are already transforming the vaccine development process. These innovations facilitate more efficient target identification, enhance vaccine design, and considerably shorten development timelines, establishing a new benchmark for combating protozoan infections. Ongoing research and investment in this field are crucial to fully leverage these powerful tools, ultimately advancing global health and reducing the impact of protozoan diseases worldwide.

The future of protozoan vaccinology is bright. Multi-valent vaccines, engineered protozoa for immunotherapy, and personalized medicine all hold immense promise for improved disease control and treatment. These approaches, when combined, could provide synergistic benefits by addressing multiple facets of disease prevention and treatment. For instance, multi-valent vaccines could enhance broad protection against diverse protozoan strains, while engineered protozoa could be tailored to boost specific immune pathways. Personalized medicine, by leveraging individual genetic and immunological profiles, may further refine these strategies, ensuring maximum efficacy and minimal side effects in diverse populations. Immuno-informatics will play a central role in these advancements by facilitating target identification, vaccine design, and optimization. Tools such as reverse vaccinology, AI-driven algorithms, and digital twin technology have already demonstrated their capacity to accelerate vaccine development and enhance precision.

Despite its promise, computational vaccinology faces certain challenges. In silico predictions require validation through laboratory experiments to ensure their accuracy and efficacy. Formulating effective vaccines from short peptide sequences predicted by computational models remains an area of active research, requiring innovations in adjuvants and delivery systems. Additionally, wider acceptance and adoption of this approach within the scientific community are crucial for its broader application. Efforts to bridge the gap between computational predictions and experimental validation are essential for realizing the full potential of this field.

Looking ahead, interdisciplinary collaboration will be a cornerstone of progress in this area. Partnerships between computational biologists, immunologists, and industry stakeholders will drive innovation and facilitate the translation of computational findings into clinical successes. Global efforts to expand access to computational tools and integrate them into existing vaccine development frameworks will also ensure that the benefits of computational vaccinology reach the populations most in need.

By uniting computational tools, immuno-informatics, and experimental science, the global health community can transform the fight against protozoan diseases. This approach not only addresses current challenges but also paves the way for innovative, accessible, and effective vaccines tailored to the unique needs of diverse populations worldwide.

## Author contributions

OH led the initial conceptualization, method development, and drafted the manuscript. IDP supervised the project, provided critical revisions, and contributed substantially to methodological refinement. Both authors contributed to formal analyses and approved the final manuscript for submission.

## CRediT authorship contribution statement

**Hashim Omar:** Writing – original draft, Formal analysis, Data curation, Conceptualization. **Isabelle Dimier-Poisson:** Writing – review & editing, Validation, Supervision, Data curation, Conceptualization.

## Declaration of Competing Interest

The authors declare that they have no known competing financial interests or personal relationships that could have appeared to influence the work reported in this review.
